# Pneumothorax, pneumomediastinum, tracheo-esophageal fistula presenting with endotracheal intubation in post-cesarean period: A case report

**DOI:** 10.1186/1757-1626-1-134

**Published:** 2008-08-29

**Authors:** Hafize Oksuz, Nimet Senoglu, Beyazýt Zencirci, Meral Ezberci, Mehmet Fatih Yuzbasioglu

**Affiliations:** 1 Kahramanmaras Sutcu Imam University Medical Faculty, Department of Anesthesiology and Reanimation, Kahramanmaras, Turkey; 2 Kahramanmaras Sutcu Imam University Medical Faculty, Department of General Surgery, Kahramanmaras, Turkey

## Abstract

**Background:**

The non-malignant, acquired tracheoesophageal fistulas (TEF), resulting from tracheal intubation are usually iatrogenic lesions. Tracheal lesions resulting from intubation may occur and pneumomediastinum, pneumothorax or subcutaneous emphysema may develop due to the stream of air.

**Case Presentation:**

We present a-39-year old, Caucasian patient, developing severe hypoxia fallowing cesarean section under general anesthesia. The findings of the patient were diffuse subcutaneous emphysema; together with pneumothorax and pneumomediastinum TEF was diagnosed in the patient by bronchoscopy and eusophagoscopy performed due to cough and difficulty in swallowing developing after extubation.

**Conclusion:**

It is important to the clinicians to be aware of the TEF can be accompanied to the traumatic intubation and urgent endoscopy or water-soluble contrast radiography may be prudent.

## Background

Pneumothorax is a pulmonary complication that occurs rarely during pregnancy and a few cases of pneumothorax, pneumomediastinum and extensive subcutenous emphysema have been reported [1].

Tracheo-esophageal fistula (TEF) formation is a rare complication of endotracheal intubation. This complication is generally thought to be iatrogenic and occurs in less than 1% of patients. High-volume, low-pressure cuffs have made TEF an infrequent occurrence; however, it still poses as a potential life-threatening condition. Acquired TEF may cause pneumomediastinum, pneumothorax or subcutaneous emphysema due to the leakage of air from trachea to the neighboring structures [2].

Here, we present a case of pneumomediastinum, pneumothorax and subcutaneous emphysema developing post-cesarean due to TEF which has been thought to be the result of traumatic intubation.

## Case presentation

A 39-year old, Caucasion, multigravida female was admitted for induction of labour at 42 weeks' gestation at obstetric unit of a peripheral hospital. In her past history, she gave birth to her five children by vaginal delivery without any complications. She was a non-smoker and had no chest disease. Artificial rupture of membranes demonstrated heavily meconium-stained liquor, and cardiotocograpy showed repetitive late decelaritons with diminished beat-to beat variability and emergency caesarean section was planned. Preoperatively she was oriented. She had pulse 115 beats/min, respiratory rate 12/min and blood pressure 160/90 mmHg. Airway assessment identified class 3 Mallampati. Examination of the chest revealed normal vesicular breathing without any added pathological sounds. As we learned from her obstetrician, following a rapid sequence induction, intubation was performed in the second attempt by using stylet with number eight low volume-high pressure endotracheal tube by a very junior medical officer and was considered difficult. General anesthesia was maintained by isoflurane end-tidal concentration 0,8–1% in oxygen and N_2_O. Although no complication was observed during the operation, she suddenly developed respiratory distress and persistent hypoxia soon after extubation. Within the next 3 min, the patient's saturation declined to 65% in spite of 100% O_2 _support, and her blood pressure dropped to 65/40 mmHg with a pulse of 158 beats/min. She was reintubated and was refered to our hospital as intubated and ventilated with transport ventilation for further investigation and management.

On the first examination, she had Glasgow Coma Scale of E_2_M_3_V_3_. Her blood pressure was 70/40 mmHg with a pulse of 145 beats/min, and her O_2 _saturation was 65% with pulse oximeter. Breath sounds was not heard on auscultation on the left hemithorax while cardiovascular examination was normal except for tachycardia. Extensive subcutaneous emphysema was noted by palpation mainly on the left side of the thorax and neck. Immediately diagnostic pleural puncture was performed and air was aspirated from the left apical region of pleura.

**Figure 1:**
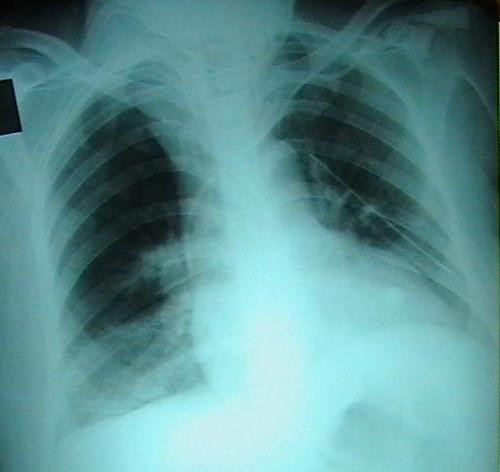
**The first X-ray graphy in the emergency service after left tube thoracostomy.** Mediastinum was enlarged in chest roentgenograms.

**Figure 2:**
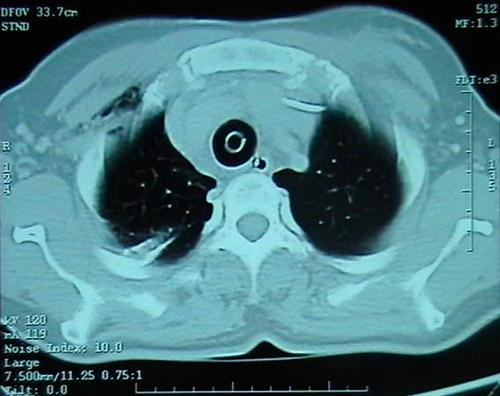
Pneumothorax regressed after tube thoracostomy.

The tube-thoracostomy was performed and 28 French sized chest tube with an under water seal drain was inserted into the chest wall at the 5th intercostal space in the left mid axillary line. A portable chest X-ray revealed the left pneumothorax together with minimal right pneumothorax, pneumomediastinum and subcutaneous emphysema. The mediastinum was enlarged and thoracostomy tube was observed to reach the left hilar region (Figure 1). Computerized tomography (CT) of the thorax revealed the presence of excessive air around cervical soft tissue, in the thorax and mediastinum (Figure 2). In the second day, her oxygenation deteriorated suddenly and it was seen on chest radiography that pneumothorax involved the right hemithorax. completely. A second chest-tube was also inserted into the right chest wall. The fiberoptic bronchoscopy was performed due to extensive atelectasia seen on chest X-ray. Bronchoscopic examination revealed diffuse hemorrhage in trachea and thrombothic plug obstructing the left bronchus almost completely. The plug was removed from the left main bronchus. Since clinical and radiological findings of pneumothorax improved, the right and left intercostals drains were removed on the fifth day. The patient was also extubated on the same day, as the oxygen saturation was persistently greater than 97% on room air. The nutrition was provided enterally with nasogastric tube until the 5^th ^day, and then she was orally fed with water and clear liquids on the first extubation day. She complained of serious throat ache and coughing during intake of food, however throat and indirect larynx examination revealed no problem explaining this condition. So, bronchoscopy and endoscopy was repeated, and TEF was diagnosed. The fistulous tract was located approximately 3 cm inferior to vocal cords, 25 cm distant from the teeth and 5–6-cm over the carina level. Following the diagnosis of TEF, percutaneous endoscopic jejunostomy was performed. Two weeks later, TEF was repaired by cervical approach. Postoperatively, the patient received parenteral nutrition for 7 days and on postoperative 9^th ^day, she had a gastrografin swallow study, which showed no evidence of TEF. The patient was discharged from the hospital on postoperative 14^th ^day.

## Discussion

The differential diagnosis of acute dyspnea in postpartum period should include pulmonary embolism caused by venous thrombosis, amniotic fluid or air embolism as well as pneumothorax and tension pneumothorax, though occurring less frequently [3]. Cases of spontaneous pneumothorax in pregnancy were rarely reported in the literature. Miguil and colleagues reported a case of spontaneous pneumothorax, pneumomediastinum and emphysema in a 19 year old primiparous patient during labour [1].

Tension pneumothorax is a life-threating condition with severe cardiorespiratory compromise. It occurs when air enters the pleural cavity on inspiration but, because of a ball valve mechanism, does not exit on expiration. This progressively enlarges the pleural space and thereby increases intrathoracic pressure. Venous return to the heart is decreased leading to reduced cardiac output and subsequently decreased blood pressure. Hypoxia results from increased shunt caused by continued perfusion of unventilated lung areas. Chest radiography should be performed if the diagnosis is unclear [3].

Risk factors for pneumothorax are respiratory infection, asthma, previous pneumothorax history, the intake of cocaine and ecstasy, insertion of central venous catheter and performing endotracheal anaesthesia with intermittent positive-pressure ventilation(IPPV). General anesthesia with IPPV for caesarean section may cause barotrauma, particularly during operation, since coughing is common when the patient is extubated fully awake [3]. Evron and colleagues[4] reported a case of bilateral pneumothorax, subcutaneous emphysema, pneumomediastinum, pneumoretroperitoneum and pneumoperitoneum detected in a 28-year-old healthy pregnant after intubation. They pointed out that this rare complication was the result of positive-pressure ventilation performed under general anaesthesia [4]. Harris presented a case with tension pneumothorax after cesarean section [5]. Acquired TEF is a rare complication of tracheal intubation, and usually results from cuff-related tracheal injury. It is known that high cuff pressure has a detrimental affect. Normal capillary perfusion pressure for tracheal mucous tissue is 20–30 cmH_2_O. If the cuff pressure is over 39 cmH_2_O, capillary perfusion in mucous tissue may cease [6].

Problems during endotracheal intubation may cause iatrogenic trauma of the upper airways [5]. The direct causes of the rupture are difficult tracheal intubation, particularly with a stylet inside the tube and overdistension of the cuff of the tracheal tube [7].

In the presented case, the difficulty of intubation, use of inappropriate stylet, use of high pressure-low volume endotracheal tube, and being a pregnant woman were the risk factors for development of TEF.

In study of Kalaud and colleagues, use of stylet in intubation in 4 of the 12 cases have been mentioned. The size of endotracheal tube and swelling of cuff may contribute to trauma. Many researchers assert that prevalence of iatrogenic tracheal rupture is higher in females and this assertion leads to conclusion that the membraneous trachea is less firm in women and children as compared with men [8].

The most common physical finding of TEF is subcutaneous emphysema in the neck or upper chest, especially during positive pressure mask ventilation, which can force gas between fascial planes into the mediastinum and subcutaneous tissue. Diagnostic methods include plain chest radiograph, which often shows subcutaneous emphysema, pneumothorax, pleural effusion, and pneumomediastenium [9]. In our patient, we detected extensive subcutaneous emphysema in the neck and left upper chest on admission. Subsequent chest X-ray revealed subcutaneous emphysema, left pneumothorax, and pneumomediastenium.

When diagnosed after extubation, the most frequent sign of TEF is coughing after swallowing. A high index of suspicion is required in patients at risk for developing a TEF. The diagnostic evaluation is by bronchoscopy and esophagoscopy [10]. Similarly, TEF was diagnosed by bronchoscopy and esophagoscopy performed due to coughing with swallowing which developed after extubation in our patient.

When the diagnosis has been made, the immediate goal should be to minimize tracheobronchial soilage by placing the cuff of a tracheostomy tube distal to the fistula. The basic aim of the treatment is to improve airway contamination and insufficient nutrition. Reflux of gastric contents is diminished by placement of a gastrostomy tube, and adequate nutrition is facilitated by inserting a jejunostomy tube. Surgical correction is required because spontaneous closure is rare, but surgery should be postponed until the patient is weaned from mechanical ventilation [10].

## Conclusion

The presence of traumatic intubation attempts is known to constitute a risk to TEF or iatrogenic injuries to trachea. We believe that, TEF observed in our case is secondary to difficult and traumatic intubation which is known as one of the risk factors for TEF. During traumatic intubation, when the possibility of esophageal injury cannot be excluded, urgent endoscopy or water-soluble contrast radiography may be prudent. Our experience confirms that early diagnosis and management is associated with a more favorable outcome.

## Abbreviations

Tracheo-esophageal fistula: TEF, Computerized tomography: CT, intermittent positive-pressure ventilation: IPPV

## Authors' contributions

HO carried out the patient's diagnosis, drafted the manuscript. NS performed the case management, drafted the manuscript. BZ and ME participated in the patient's management. MFY participated in the writing of the case report. All authors read and approved the final manuscript.

## Consent

Written informed consent was obtained from the patient for publication of this case report and accompanying images. A copy of the written consent is available for review by the Editor-in-Chief of this journal"
